# Elevated Donor‐Derived Cell‐Free DNA in Heart Transplants Beyond 5 Years: Early Warning or False Alarm?

**DOI:** 10.1161/JAHA.126.052197

**Published:** 2026-09-09

**Authors:** Juka S. Kim, Erik Henricksen, James Pak, Yasbanoo Moayedi, Jeff Teuteberg, Hannah Valantine, Kiran K. Khush

**Affiliations:** ^1^ Division of Cardiovascular Medicine Stanford University Palo Alto CA USA; ^2^ Department of Transplant Stanford Healthcare Palo Alto CA USA; ^3^ University Health Network Toronto ON Canada; ^4^ CareDx Brisbane CA USA

**Keywords:** donor‐derived cell‐free DNA, graft dysfunction, heart transplantation, Heart Failure, Cardiomyopathy, Precision Medicine, Transplantation

Acute rejection remains a serious risk following heart transplantation (HT). Endomyocardial biopsy has historically been the cornerstone of rejection monitoring. However, it is invasive, samples are only limited to myocardial regions, is subject to interobserver variability, and imposes a substantial burden on patients.^1^ Because endomyocardial biopsy evaluates localized tissues, it may fail to capture the full burden of rejection, which can be patchy and heterogeneous within the allograft.

Accordingly, noninvasive blood‐based biomarkers have emerged as alternative surveillance tools, beginning with gene expression profiling for acute cellular rejection in 2010 and more recently donor‐derived cell‐free DNA (dd‐cfDNA) for both acute cellular rejection and antibody‐mediated rejection (AMR) surveillance in 2017.^1^, ^2^ Successful and durable outcomes after HT rely heavily on routine surveillance and close follow‐up.^3^ Although dd‐cfDNA has been increasingly adopted for rejection monitoring in the early post‐HT period, its role beyond 5 years remains poorly defined.

To address this gap, we conducted a single center, retrospective analysis of consecutive adult (aged ≥18 years) recipients of isolated HT between April 3, 2008, and August 24, 2020, who underwent dd‐cfDNA testing beyond 5 years post‐HT as part of routine annual testing. For patients with multiple measurements, the highest dd‐cfDNA value was used for analysis. Associations between dd‐cfDNA levels and time since HT, as well as graft function assessed by left ventricular ejection fraction (LVEF) on routine echocardiogram obtained within 1 year of testing were evaluated using linear regression analysis. To account for heteroskedasticity, statistical significance was assessed using permutation tests. Graft dysfunction was defined as LVEF <50% and compared with preserved LVEF (≥50%) using Chi‐square testing and Fisher exact test as appropriate. The study was approved by the Stanford University Institutional Review Board, and all patients provided informed consent. The data that support the findings of this study are available from the corresponding author upon reasonable request.

Among 108 HT recipients with dd‐cfDNA measurements, mean age was 52±12 years, 78 (72%) were men, 65 (60%) were White, and 91 (84%) were transplanted for non‐ischemic cardiomyopathy. Most recipients (93%) received induction therapy with antithymocyte globulin. The majority (87%) were transplanted before October 2018, 53 (56%) of whom were listed as Status 1A. Time since HT ranged from 5 to 16 years (mean 8.3±2.7 years) (Figure). Median dd‐cfDNA was 0.07% (interquartile range 0.04–0.50), with 34 (31%) values ≥0.20%.

**Figure 1:**
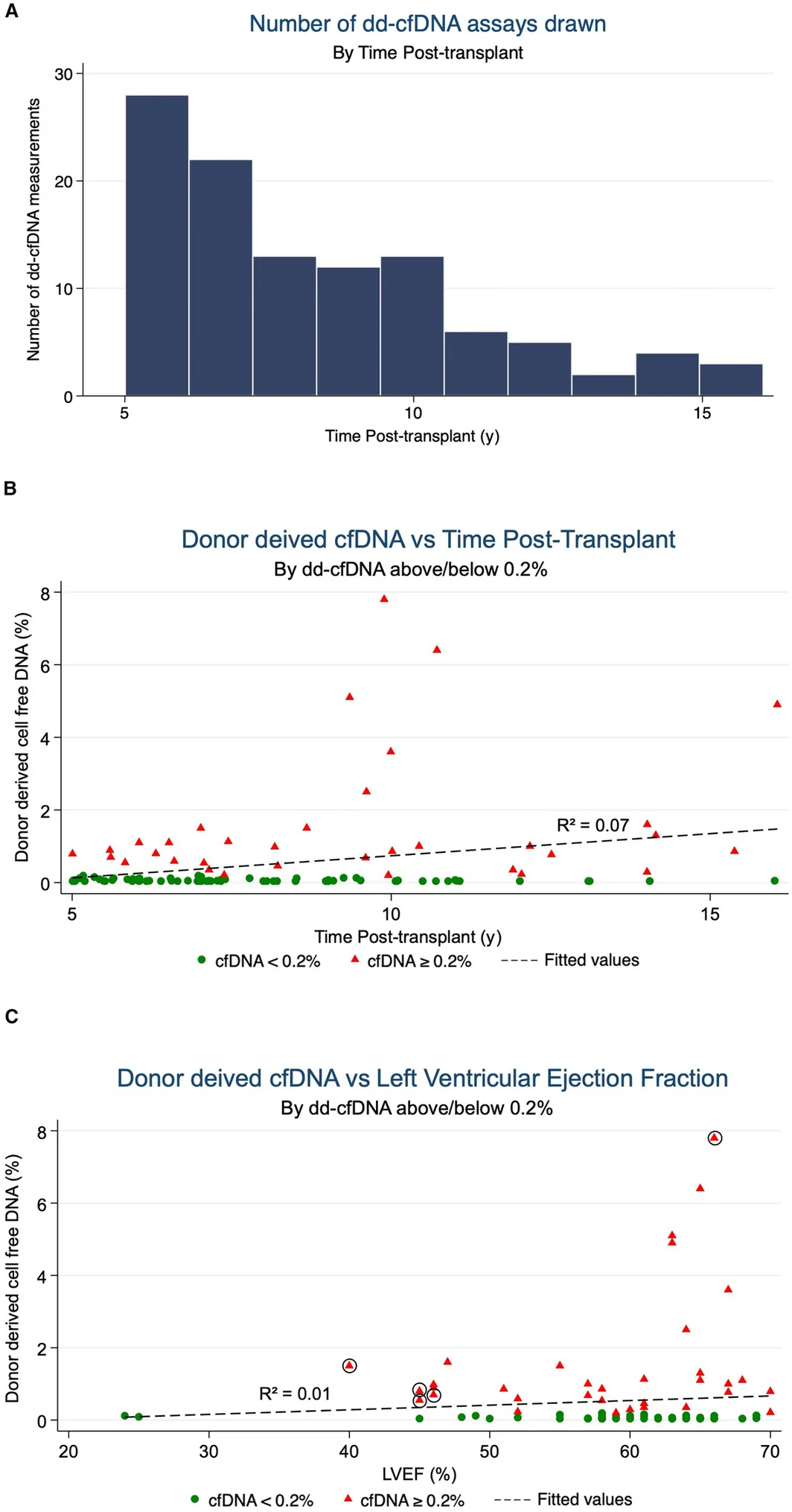
dd‐cfDNA association with time post‐HT and LVEF. **A**, Number of dd‐cfDNA assays drawn, by time post‐HT. **B**, Donor‐derived cell‐free DNA levels, by time post‐HT. **C**, Donor‐derived cell‐free DNA levels, by LVEF. Circled in panel C represents HT recipients with elevated dd‐cfDNA and acute antibody‐mediated rejection or cardiac allograft vasculopathy. dd‐cfDNA, donor‐derived cell‐free DNA; HT, heart transplantation; and LVEF, left ventricular ejection fraction.

There was a statistically significant but weak positive association between dd‐cfDNA and time since HT (R^2^=0.07, β<0.01, *P*=0.01), whereas there was no statistically significant association between dd‐cfDNA and LVEF (R^2^=0.01, β=0.01, *P*=0.40) (Figure). An LVEF <50% was coincident with 12 (11%) dd‐cfDNA measurements. Graft dysfunction was more frequent among patients with dd‐cfDNA ≥0.20% compared with those with lower levels (21% [7/34] versus 7% [5/74], *P*=0.048). In patients with elevated dd‐cfDNA, graft dysfunction was attributed to acute AMR (29%), new cardiac allograft vasculopathy (CAV) (43%), history of recurrent AMR (14%), and history of severe CAV (14%). In contrast, graft dysfunction in patients with low dd‐cfDNA levels was seen in chronic systolic dysfunction, with majority (80%) in the setting of angiogram negative/intravascular ultrasound positive (maximal intimal thickness >0.5 mm) CAV or biopsy negative rejection. Notably, all patients with very elevated dd‐cfDNA levels (>2.00%) had a normal LVEF. This included 6 individuals with values ranging from 2.50% to 7.80%. In most cases, the cause was unclear and presumed to reflect prior CAV or acute cellular rejection/AMR. One of these patients (dd‐cfDNA 7.80%) had asymptomatic, persistent AMR requiring treatment.

Beyond 5 years post‐HT, most recipients had low dd‐cfDNA levels and normal LVEF during routine surveillance. Although dd‐cfDNA levels were not significantly associated with LVEF, elevated levels were associated with a 3‐fold higher prevalence of graft dysfunction. In these patients, dysfunction was most often linked to AMR or new CAV, whereas those with low dd‐cfDNA and reduced LVEF typically had chronic, previously established systolic dysfunction.

This study is limited by small sample size and should therefore be considered hypothesis‐generating. Recipients surviving longer may undergo less frequent surveillance testing, which could limit direct comparisons across post‐transplant duration and introduce survivorship bias. Additionally, the relationship between dd‐cfDNA, time post‐HT, and LVEF may be non‐linear, and cumulative or episodic injury may be more relevant than absolute time. Because routine endomyocardial biopsy, gene expression profiling, and donor‐specific antibody testing were not performed beyond 2 years post‐HT, we were unable to compare dd‐cfDNA with these modalities in the long term. Lastly, patients diagnosed with rejection after elevated routine dd‐cfDNA screening often underwent additional surveillance testing, possibly resulting in more dd‐cfDNA measurements. However, dd‐cfDNA levels generally declined after treatment, making the initial screening value typically the highest measurement.

Recent studies have demonstrated increased mortality risk among HT recipients with elevated dd‐cfDNA despite negative endomyocardial biopsy within the first 2 years post‐HT,^4^, ^5^ underscoring its value for early risk stratification. Our findings suggest a potential role for dd‐cfDNA in long‐term risk stratification beyond 5 years, particularly given that patients with the highest dd‐cfDNA levels maintained normal graft function.

## Sources of Funding

None.

## Disclosures

JT is an employee of CareDx. KK is on the advisory board for CareDx and consults for Broadview Ventures.

## Supporting information

AHA Journals Disparities Guidelines Checklist

STROBE Statement
