# Supplementary material for: Elevated Donor‐Derived Cell‐Free DNA in Heart Transplants Beyond 5 Years: Early Warning or False Alarm?
Source: J Am Heart Assoc. 2026 Sep 9;15(19):e052197. doi: 10.1161/JAHA.126.052197 (PMC13744994; doi:10.1161/JAHA.126.052197)
Supplement: Supplementary file 1 [file JAH3-15-e052197-s001.pdf]

**AHA Journals Racial and Ethnic Disparities Reporting Guidelines**			
**Section/Topic**	**Item No.**	**Recommendation**	**Provide page # in text / Confirm that this was "done throughout" / N/A**
**Introduction**			
	1a	Discuss a framework (e.g., conceptual model) for studying race and/or ethnicity in this context	N/A
	1b	Discuss social and structural forms of racism and/or bias	N/A
**Methods**			
Categorization of race/ethnicity	2a	Describe categorization of race and ethnicity (e.g., self-identification)	N/A
	2b	If race and ethnicity are codified by others, be specific in conveying how the categories were attributed	N/A
	2c	Describe potential limitations of existing data sources	N/A
Terminology			
	3a	Capitalize race and ethnicity terms and use as adjectives rather than nouns (e.g., "Black patients," not "blacks", "White patients," not "whites", etc.)	Done throughout
	3b	Describe specific racial and ethnic makeup of smaller population groups; when possible avoid "non-White" or "Other"	Done throughout
	3c	Use accurate terminology: Hispanic and Latino/a/-x are ethnicities; the term "White" is preferred over Caucasian	Done throughout
Analyses			
	4a	Provide context and analytical use of race/ethnicity as a covariate in risk-adjustment models	N/A
**Results**			
	5a	Avoid statements of causal inference or culpability (e.g., "Black adults did not respond to *x* medication." Instead, "Among Black adults, *x* medication was less effective")	N/A
**Discussion**			
	6a	Describe the relevant structural and social factors that influence the study question	N/A
	6b	Avoid using genetics in isolation to explain social constructs	N/A
